# Infection prevention and control during COVID-19 pandemic: realities from health care workers in a north central state in Nigeria

**DOI:** 10.1017/S0950268821000017

**Published:** 2021-01-07

**Authors:** O. S. Ilesanmi, A. A. Afolabi, A. Akande, T. Raji, A. Mohammed

**Affiliations:** 1 Department of Community Medicine, University of Ibadan, Ibadan, Oyo State, Nigeria; 2 Department of Community Medicine, University College Hospital, Ibadan, Oyo State, Nigeria; 3 Africa Centers for Disease Control and Prevention, Addis Ababa, Ethiopia

**Keywords:** Coronavirus, COVID-19, health care workers, infection prevention and control, Nigeria

## Abstract

Health care workers (HCWs) are vulnerable to the risk of infections and could become vectors of onward transmission of coronavirus disease 2019 (COVID-19). Little is known about the factors which could contribute to increased COVID-19 infection among HCWs in Nigeria. We aimed at assessing the causes of COVID-19 infection among HCWs. We used a qualitative study design to conduct in-depth interview among 16 frontline HCWs participating in the COVID-19 response in Kwara State, Nigeria. Colaizzi's phenomenological method was used in the qualitative analysis of data. We found that HCWs were aware of their vulnerability to the COVID-19 infection, and the reasons attributed included poor knowledge of IPC measures for COVID-19, inadequate supply of personal protective equipment (PPE), poor political will and inadequate health facilities (HFs) management support. Improved political will and better involvement of HFs management teams in infection prevention and control (IPC) systems are needed to reduce the risk for COVID-19 infection among HCWs. We recommend scale-up training on IPC measures particularly hand washing and use of PPE as well as the development of effective points of care risk assessment with a high index of suspicion in HFs.

## Introduction

The development of vaccines and medical research for the novel coronavirus disease (COVID-19) is ongoing, however, the demand for regular provision of health care keeps rising [1]. The provision of quality health care during the COVID-19 pandemic depends largely on the health of health care workers (HCWs). This group of persons are especially vulnerable to the risk of infections and could become vectors of onward transmission of COVID-19 [1, 2]. The rapidly evolving pandemic has overwhelmed the health system and has burdened HCWs and health facilities (HFs) both globally and locally [3]. HCWs face the stress of physical and mental exhaustion, the pain of losing patients and loved ones and difficulties in making triage decisions [1, 4]. A resulting implication is an increased pressure on the global health workforce [5]. The current COVID-19 pandemic emphasises the need for every HF to have critical-care HCWs in sufficient numbers and with adequate skills [6].

Globally, occupational exposure account for about 40% of infections among HCWs [7]. Reports from Euro News and Ripples reiterate that infection rates for COVID-19 among HCWs is estimated as 6% globally and 3% in Nigeria [8, 9]. The rise in these cases could be due to the asymptomatic nature of majority of COVID-19 cases. Similarly, infection rates for Middle East respiratory syndrome (MERS) accounted for 13.44% among HCWs in South Korea [10]. Retrospective analysis based on epidemiological data reveals that the case fatality rate (CFR) for MERS among HCWs is 5.78% [11]. The novelty, high infectious rates and associated fatality differentiates the novel coronavirus from other infectious illnesses with which the human race has been faced. For the prevention of COVID-19, safety measures such as hand hygiene, social distancing and the use of personal protective equipment (PPE) were put in place [6].

Safety guidelines have been developed by the Nigerian Centre for Disease Control (NCDC) in line with recommendations from the World Health Organization (WHO) regarding the coronavirus [6]. Existing evidence suggests that COVID-19 is a viral infection transmitted by droplets and contact, rather than by air [6]. This explains the existence of precautions on social and physical distancing, environmental hygiene, as well as infection prevention and control (IPC) practices [6]. In tandem with existing recommendations from health agencies, a dire need for IPC materials, including face masks, protective gloves, gowns, face shields and respirators exists among HCWs and in health care facilities [1, 6].

Evidence from the severe acute respiratory syndrome (SARS) outbreak and its response revealed the modification of IPC procedures in adaptation to the substantial change in working conditions [6]. This necessitated the discouragement of gatherings of HCWs within and outside the hospital premises. Compulsory use of face masks was also encouraged, and physical meetings gave way to meetings on electronic media [12]. Despite the little risk of infection to the general public during this period, HCWs were particularly vulnerable due to the maintenance of close contact with patients, direct contact with droplets from patients, and inadequate supply of PPE [13].

Similarly, standard IPC precautions have existed regarding hospital-acquired infection (HAI) [14]. HCWs exist as vectors in the patient-to-patient transmission of HAI. Available evidence demonstrates the effectiveness of safety guidelines including the use of PPE, disinfection of equipment and environment and waste management. However, compliance to IPC measures remain poor [14]. The reasons accrued to non-compliance included lack of knowledge, poor risk assessment and behavioural change and inadequate supply of PPE. A study of methicillin-resistant *Staphylococcus aureus* or vancomycin-resistant *Enterococcus* patient transfers to the Radiology department in a Metropolitan hospital also confirmed that infection control measures were not adopted by HCWs especially when the infection status of patients was unknown [15].

The level of adherence of Nigerian HCWs to IPC measures on COVID-19 remains unknown. To the best of our knowledge, the existence of certain factors which could contribute to increased COVID-19 infection among HCWs in Nigeria has not been examined. A dive into research among HCWs could elicit key information necessary to reduce further infection of COVID-19 among HCWs. Hence, this study aimed at an assessment of the causes of COVID-19 infection among HCWs and the solutions.

## Methods

### Research design

We conducted a qualitative study on frontline HCWs involved in the COVID-19 response in Kwara State, Nigeria. The choice of qualitative study design was made to be able to document a broad range of experiences of HCWs; therefore, the phenomenology method was used to underpin the study.

#### Personal characteristics

As of the time of the interview, the researchers were members of the COVID-19 outbreak response team in Nigeria. Two of the authors; OSI and AAA, served as interviewers during the interview. To overcome gender-bias, the interviewers were male and female. Both interviewers, had previous training in qualitative research methodology. One of the interviewers is a medical doctor with master's degree in public health, while the second was currently enrolled in the master's of Public Health programme. The interviewers were previously members of an interviewing team for another completed qualitative research among COVID-19 positive HCWss in another state in Nigeria. However, both interviewers were not part of the supervisors of the frontline HCWs.

#### Relationship with participants

We approached HCWs, all of whom provided consent for participating in the study mainly because one of the interviewers was known to them. Prior to the commencement of the study, one of the interviewers had attended the same training with the study participants. Therefore, this helped to gain participants confidence and build a co-workmate relationship among the interviewers and the study participants. The study participants were informed that the study aimed at identifying gaps that required attention to reduce their vulnerability to COVID-19.

#### Participant selection

Purposive sampling was used to select the study participants. The inclusion criterion was all frontline HCWs participating in the COVID-19 response at the primary, secondary or tertiary levels of care. We included physicians and nurses only to generate sufficient information on the experiences of frontline HCWs who were directly providing care to COVID-19 patients. The interview method was adapted for greater privacy and confidentiality in exploring individual views and in-depth information. Also, the sensitivity of COVID-19 especially among HCWs contributed to the use of the interview method in a designated setting which assured of privacy. Sequel to obtaining informed consent, we scheduled and commenced face-to-face in-depth interviews within seven days (22nd to 28th June 2020) among HCWs in Kwara State, Nigeria. We continued to interview the HCWs who met the inclusion criteria, but however limited the sample size to 16 frontline HCWs (12 doctors and 4 nurses) when saturation; a point where no new information was gotten from the study participants, was reached.

#### Setting

Data were collected at a relaxation centre close to the Ministry of Health in Ilorin, Kwara state. Each interview session lasted between 30 and 40 min, and no other person was present at the interview site besides the interviewer, the research assistant and each interviewee.

#### Data collection

Informed consent was obtained from each participant to record the interview. Due to the busy schedule of the interviewee and the nature of their job one session of repeat interview was conducted by the interviewers to verify the opinions of the HCWs who had participated previously in the study. Both sessions of interviews were audiotaped, transcribed verbatim and harmonised by all the authors to ensure the effective communication of respondents' experiences. The field notes taken during the interview also helped to appropriately record recurring statements and facial expressions of the frontline HCWs. Each transcript was returned to each study participant for additional comments and/or correction(s).

### Interview guide

An interview guide was developed to guide interviewers during the discussion. This included questions like: What do you know about IPC in the management of COVID-19 patients? Why are more HCWs contracting COVID-19? Any recommendation to reduce the infection rates among HCWs? Piloting of the questions on the interview guide was done among frontline HCWs at secondary HFs in Oyo State, Southwest Nigeria to test for ambiguity. Ambiguous questions were rephrased and simplified. During the interview, prompts such as ‘Could you please explain further’ ‘Could you clarify?’ and ‘Please tell me more about that’ were used to enhance the depth of the discussion.

### Data analysis

The Colaizzi's phenomenological method was used in the qualitative analysis of data. This method is dependent on rich first-person accounts of experience through face-to-face interviews, written narratives, online interviews etc. Descriptive phenomenology enhances a detailed understanding of the subject being investigated and is commonly used in health research [16]. This qualitative method sticks to and analyses data using seven vital steps without losing any element of the data. They include familiarisation of the researcher with the data, identification of relevant significant statements, formulation of meanings, development of cluster themes, description of the phenomenon while incorporating all themes, development of the fundamental structure, and its verification from few participants [17]. Manual transcription of each interview was done by three authors (OSI, AAA and AA), after which themes were identified from the data by AAA and OSI. For the verification process, three participants were invited for an interview which was conducted to ascertain if the identified themes fully matched their contributions on the factors contributing to increasing COVID-19 infection rates among HCWs and recommendations.

### Ethical review

Ethical approval in accordance with the Helsinki Declaration was obtained from the Nigerian Institute of Medical Research (IRB/20/048). Also, verbal informed consent was obtained from all participants, and confidentiality of information was guaranteed prior to the commencement of the interview.

## Results

**Fig. 1.**
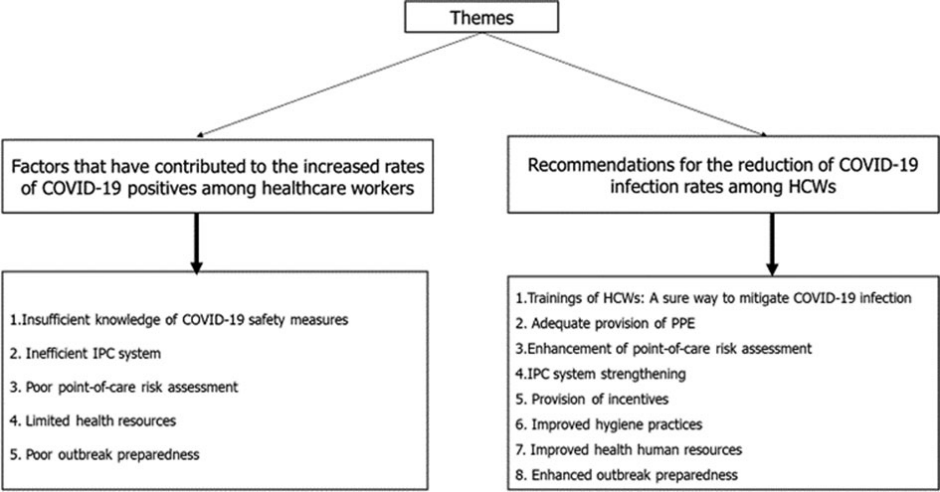
Identified themes from the in-depth interview conducted among COVID-19 frontline HCWs.

Sixteen HCWs (4 nurses and 12 physicians) were interviewed; 3 (18.75%) females and 13 (81.25%) males. Other socio-demographic characteristics of the HCWs interviewed are shown in Table 1. Figure 1 shows the identified themes and sub-themes from the in-depth interview.

**Table 1.** Sociodemographic characteristics of HCWs interviewed

Profession	Age (years)	Sex	Highest level of education	Years of practice	Cadre
Nurse 1	35	Female	BSc	8	Senior nursing staff
Nurse 2	52	Male	BSc	10	Nurse
Nurse 3	54	Male	BSc	27	Nurse
Nurse 4	58	Male	PhD	25	Senior lecturer
Physician 1	28	Male	MBBS	5	Resident doctor
Physician 2	33	Male	MBBS	9	Senior medical officer
Physician 3	36	Female	MBBS	9	Private employee
Physician 4	37	Male	Post-graduate	10	Medical officer
Physician 5	37	Male	Post-graduate	10	Principal medical officer
Physician 6	39	Male	Post-graduate	13	Principal medical officer
Physician 7	40	Female	Post-graduate	8	Medical officer
Physician 8	40	Male	Post-graduate	12	Principal medical officer
Physician 9	43	Male	Graduate	16	Consultant
Physician 10	48	Male	Graduate	10	Senior medical officer
Physician 11	49	Male	Post-graduate	10	Principal medical officer
Physician 12	60	Male	Post-graduate	31	Medical director

### Sub-theme one: Insufficient knowledge of COVID-19 safety measures

Among the study participants, two females (12.5%) and five (31.25%) males stated that poor knowledge of IPC measures for COVID-19 as a factor which could increase COVID-19 infection rates among HCWs (Table 2).

‘I am certain that many healthcare workers do not possess adequate knowledge of IPC in managing COVID-19 patients. This will therefore put many of us at risk of contracting COVID-19.’ (**N2)**

The poor knowledge of IPC was viewed among respondents as a consequence of the lack of sufficient IPC training. IPC training is meant to equip HCWs for the prevention of COVID-19. However, when such training is inadequate, HCWs are increasingly posed with the risk of COVID-19 infection.

‘IPC trainings prepare Health Care Workers for risk prevention while providing care for COVID-19 patients, but when trainings are inadequate and irregularly scheduled, we cannot overcome COVID-19 infection among health providers…’ (**N1)**

Many respondents to the poor or inappropriate use of PPE among HCWs. Although PPE were not always available, HCWs do not appropriately use them during the limited time in which they were available.

‘PPE are not always adequate and available for use.’ **(N2)**

‘A lot of healthcare workers do not use PPE in the manner in which it has been stipulated for use…’ **(N1)**

‘Donning and doffing are poorly done among many health workers. Many of us do not possess sufficient knowledge of these basic IPC measures.’ **(P1)**

Deliberate disregard of IPC measures has also contributed to the increasing rates of COVID-19 infection among frontline HCWs who manage COVID-19 patients. Many HCWs who are religious folks deny the existence of COVID-19, and thus fail to comply with recommended IPC measures.

‘Non-adherence to standard safety precautions have been observed among many health workers. They are unknowingly putting their lives at risk…’ **(P4)**

‘The overbearing influence of religious beliefs wrongly influence the decisions of many health workers regarding IPC compliance’. (**P2)**

**Table 2.** Respondents' contributions under the various themes and sub-themes

Theme	Sub-themes	Summary
Knowledge on COVID-19 safety measures	IPC	Many HCWs have poor knowledge of IPC Lack of IPC skills Non-adherence to standard safety precautions
	PPE usage	Poor knowledge on donning and doffing Inappropriate use of PPE Lack of adequate PPE Poor attitude and poor practices of PPE usage Many HCWs deny the existence of COVID-19, and so have poor knowledge of its safety measures
IPC system	Weak IPC system	HFs management's overbearing bureaucratic methods Exclusion of management from the IPC system
Point-of-care risk assessment	Poor risk assessment	Poor triaging processes among many HCWs Inadequate triaging equipment Inadequate knowledge of patient triaging Negligence of HCWs Many HCWs do not handle patients as suspects once they are asymptomatic Poor index of suspicion among HCWs Undisclosed and incomplete medical and travel history from patients and relatives
Resources for health	Inadequate human resources for health	Increased work load for HCWs Working under much pressure could increase vulnerability Poor financial motivation Absence of break periods Inadequate IPC tools Poor water supply at facilities Poor handwashing practices Environmental pollution Poor compliance to standard preventive measures
Outbreak preparedness	Poor outbreak preparedness	Inadequate logistics Lack of training on infectious disease management Poor political will
Recommendations	Training	Training on PPE usage and general overview of IPC We need IPC training and IPC drilling at HFs Training on screening, isolation and notification of confirmed cases Increased health education on the use of face masks and shield Immediate screening and isolation of suspected cases Training on management of infectious diseases
	Adequate provision of PPE	Encouragement on the proper use of PPE (donning and doffing) Provision of adequate PPE Improved stewardship from management on rational use of PPE Provision of PPE and sanitation kits Enforcement of PPE compliance
	Point-of-care risk assessment	Handling of all patients as suspects for COVID-19 Prompt commencement of patients' care Adequate supply of triaging equipment
	IPC system strengthening	Establishment of IPC team where none exists Proper incorporation of management into IPC team Institutionalisation of IPC in quality improvement plans of hospitals Establishment of IPC champions and cough officers in all departments Proper monitoring and supervision Capacity building for HCWs The need for IPC focal persons in each facility
	Provision of Incentives	Provision of incentives and rewards Availability of financial motivation Provision of hazard allowance commensurate to risks borne
	Improved sanitation	Frequent handwashing with soap and water Regular fumigation of the hospital premises Regular water supply Enforcement of hand hygiene Improved availability of sanitation kits
	Increased human resources for health	Recruitment of more health professionals Reduction of work load for frontline HCWs Inclusion of break periods during duty hours
	Enhanced outbreak preparedness	Provision of adequate logistics Regular supply of up-to-date information on COVID-19 Improved government support

### Sub-theme two: inefficient IPC system

The exclusion of HFs management teams from IPC committees have contributed to the increased rates of COVID-19 infection among HCWs. The overbearing bureaucratic methods of the management teams at HFs could have hindered the availability of IPC materials in adequate measures for use by HCWs. Lack of IPC focal points in HFs has contributed to inefficient IPC system. Overall, inefficient IPC system was stated by 1 (6.25%) each among male and female participants.

‘Management teams of HFs strictly control the use of IPC materials. Are these materials not supposed to be released for our safety?’ **(N3)**

‘When hospital management teams are not included in the IPC committee, how would they know our challenges, and proffer timely solutions?’. **(P7)**

### Sub-theme three: poor point-of-care risk assessment

The unavailability of adequate triaging equipment and poor triaging process has been a factor responsible for the increased number of HCWs who have tested positive for COVID-19. When poor index of suspicion exists among HCWs, risk assessment of patients cannot be properly conducted. Incomplete disclosure of medical history from many patients and relatives could have made HCWs throw caution to the wind while caring for these patients who may have been infected with COVID-19. Poor point-of-care risk assessment was described as a contributory factor to increasing infection rate among HCWs by three (18.75%) participants.

‘Many healthcare workers do not have high index of suspicion.’ **(P5)**

‘Some caregivers lie to healthcare providers. They will deliberately conceal the truth regarding the exposure of their relatives to confirmed COVID-19 cases’. **(N1)**

‘Lateness in the diagnosis of COVID-19 positive patients is a major challenge…’ **(P10)**

### Sub-theme four: limited health resources

Poor financial motivation and increased workload have also accounted for the increased COVID-19 infections among HCWs. In instances of shortage of PPE, HCWs are not encouraged to invest on the purchase of PPE from the meagre remuneration received as members of the COVID-19 response team. Increased workload was described as a reason for in forgetfulness regarding PPE usage by two (12.5%) participants e.g. face masks among many HCWs.

‘Long working hours are challenging to the regular use of PPE, some of which obstruct normal breathing.’ **(P6)**

‘The incentives are meagre, and do not encourage self-purchase of PPE.’ (**P8)**

The lack of IPC tools was described in many health care facilities by two (12.5%) participants. The lack of these lifesaving tools will pose threats to the safety of HCWs against COVID-19 while providing care to COVID-19 positives. The availability of soaps, hand sanitisers in inadequate quantities and irregular water supply also compromise the protection of HCWs against COVID-19 infection.

‘Regular water supply is not available for use…’ (P3)

‘IPC tools are currently inadequate. This will not assure of our safety …’ (N4)

### Sub-theme five: poor outbreak preparedness

Among the respondents, inadequate logistics, lack of training on infectious disease management, poor political will and poor behavioural change were stated among two (12.5%) males and one (6.25%) female as factors which have increased COVID-19 infection rates among HCWs. There is a need to change behaviour due to non-compliance despite presence of IPC materials.

‘We lack adequate information and logistics on the management of COVID-19 cases. Management modalities were changed too frequently.’ **(N4)**

‘Poor political will has not helped the provision of resources needed by healthcare workers who are involved in the COVID-19 response.’ **(P9)**

‘Many of us had not received training on infectious disease management prior to the outbreak of COVID-19’. **(P12)**

### Theme two: recommendations

#### Sub-theme one: training of HCWs: a sure way to mitigate COVID-19 infection

Training should be focused on the use of PPE and IPC measures, with the improvement of health education among HCWs across all HFs. The need for increased frequency of training on PPE usage, heightening of screening, isolation and notification activities among HCWs and behavioural change was stated by 1 (6.25%) each among males and females. Behavioural change communication (BCC) cannot be overemphasised due to non-compliance despite presence of IPC materials.

‘Training of healthcare workers on IPC measures and appropriate donning and doffing is needed’. **(N1)**

‘Capacity building for healthcare workers on screening, isolation, and notification activities is highly required’. **(P11)**

#### Sub-theme two: adequate provision of PPE

Alongside the provision of PPE and sanitation kits, two male (12.5%) participants reiterated the need to encourage PPE usage among HCWs. The rational use of PPE has been suggested to depend on improved stewardship from the management of HFs, and this needs to be incorporated into tasks related to the COVID-19 reduction compliance needs to be enforced.

‘Hospital management teams should provide PPE to health facilities and should ensure compliance to outlined COVID-19 safety measures.’ **(P3)**

‘Government authorities and non-governmental organizations should make PPE more available to healthcare workers. They should not make empty promises.’ **(P9)**

#### Sub-theme three: enhancement of point-of-care risk assessment

In the management of COVID-19, HCWs need to enhance their suspicion index by classifying every patient as suspected COVID-19 cases. Also, the need for prompt commencement of care immediately confirmation of cases is ascertained was noted by 3 (18.75%) males. Also, triaging equipment need to be supplied in adequate quantities.

‘Point-of-care assessment must be enhanced among healthcare workers.’ **(P7)**

‘Triaging should be commenced immediately patients are presented to healthcare workers.’ **(P3)**

‘All patients must be treated as possible COVID-19 cases until proven otherwise.’ **(P10)**

#### Sub-theme four: IPC system strengthening

In facilities where none exists, IPC system needs to be set up, with the proper incorporation of the management teams of HFs into the IPC team. Institutionalisation of IPC in quality management plans of hospitals needs to be done, alongside the establishment of IPC champions and cough officers in all departments were described as needful by 1 (6.25%) each of male and female participants. One (6.25%) male participant suggested the need for the existence of IPC focal persons in each HF, with proper monitoring and supervision by the HF management teams.

‘The institutionalization of IPC in quality improvement plans of hospitals is highly required.’ **(P11)**

‘IPC focal persons are needed in each health facility and are to be monitored by the management teams in health facilities.’ **(N3)**

‘Management teams of health facilities should be adequately incorporated into IPC committees. Cough officers should also be assigned to each department.’ **(P1)**

#### Sub-theme five: provision of incentives

The provision of financial rewards and incentives were frequently suggested by respondents. These allowances should be commensurate to the risks that HCWs bear while caring for COVID-19 patients. Incentives and/or hazard allowance was noted as tools which prompt higher levels of commitment to work among HCWs by 1 (6.25%) each among males and females.

‘Hazard allowance should be regularly paid, and more incentives should be included for healthcare workers who are involved in the COVID-19 response.’ **P3**

‘Incentives must be provided for all categories of healthcare workers, not only those in the frontline…’ **N3**

#### Sub-theme six: improved hygiene practices

Frequent handwashing practices using soap and water and regular fumigation of hospital premises are required in the reduction of infection rates among HCWs. Improved availability of sanitation kits across all sections of HFs were also suggested by 1 (6.25%) each of males and females.

‘Healthcare workers should frequently wash their hands with soap under running water, and compliance to hand hygiene must be enforced.’ **P1**

‘Regular cleaning of the hospital premises and possible fumigation should be encouraged. Sanitation kits are to be present in all wards of each health facility.’ (**N4)**

#### Sub-theme seven: improved health human resources

The recruitment of more health professionals reduces the workload on frontline HCWs in COVID-19 management and reduce their vulnerability. The inclusion of break periods during duty hour reduces the stress on HCWs, and enhance proper decision-making regarding risk assessment, stated two male (12.5%) participants.

‘More healthcare workers should be recruited into the COVID-19 response, and workload should be reduced.’ **(P12)**

‘Break periods must be sufficiently incorporated into duty schedules to reduce the stress faced by healthcare workers.’ **(P5)**

#### Sub-theme eight: enhanced outbreak preparedness

The provision of adequate logistics is the responsibility of both government and the hospital management in the enhancement of any outbreak preparation. The regular supply of up-to-date COVID-19 information is required to intimate HCWs of the increased risk associated with COVID-19 exposure. The need for concrete and enhanced outbreak preparedness was described by two (12.5%) males.

‘Outbreak preparedness need to be enhanced to reduce the risk associated with the exposure of healthcare workers to COVID-19.’ **(P4)**

‘Adequate logistics should be provided for all healthcare workers.’ **(P10)**

## Discussion

This study aimed at assessing HCWs regarding IPC measures for COVID-19. HCWs possess a spirit of dedication to the service of humanity and are always willing to provide the best care regardless of the prevailing circumstances. In this study, we found that the inadequate supply of PPE could increase the infection risk for COVID-19 among HCWs. Errors in the donning and doffing of PPE were also identified as contributory to COVID-19 among HCWs in Nigeria. Similar errors were noted in the West African Ebola outbreak, and this necessitated the new interim guidance during this period [18].

Although regular PPE usage has been a source of physical distress to HCWs, its provision in inadequate quantities denies these HCWs of their rights to protection against COVID-19 [19]. Due to this factor, many HCWs have developed anxiety and apprehension of the fear of infection over the news of an infected colleague with whom they have had contact. These findings confirm the notions of respondents in this study regarding the dire need for adequate supplies of PPE for every HCW. This implies that the management of public health emergencies such as COVID-19 entails prioritising the health of HCWs so that they could be empowered to provide quality health services to all individuals.

The findings from this study suggest that poor knowledge of IPC or its negligence amongst HCWs is a contributory factor to the increased prevalence of COVID-19 infection among HCWs. During the SARS outbreak, poor knowledge of IPC was associated with increased levels of emotional distress and fear of infection among nurses [13]. In this study, respondents recommended BCC and enforcement of IPC measures across all facilities as solutions to reducing COVID-19 infection among HCW. Similarly, previous studies on MERS and SARS suggest that sound infection prevention measures such as adherence to the use of PPE and regular environmental decontamination was strictly enforced [6, 19].

A recent study on the experience of frontline HCWs during the COVID-19 outbreak in China suggest that non-essential HCWs were denied entry into the COVID-19 isolation wards [20]. This proactive measure was in line with sound knowledge of IPC in such facilities [20]. Hence, an improvement in the knowledge and practice of physicians and nurses through health education sessions would serve to protect physicians, nurses and their families from the COVID-19 infection.

The WHO has defined eight pillars for an IPC structure, namely: IPC programmes, IPC guidelines, education and training and surveillance. Others include multimodal strategies, monitoring and feedback, workload, staffing and bed capacity, and built environment, materials and equipment for IPC at HFs [21]. We found from this study that the existence of weak IPC structure in HFs cripples the safety of HCWs. Physicians and nurses interviewed in this study attributed long working hours during the COVID-19 outbreak as risk factors to COVID-19 infection among HCWs, as similar to findings from previous research [22, 23]. This might be due to the lack of rest, long-term exposure of HCWs to COVID-19 cases, and working under pressure.

The success of any IPC structure hinges on the inclusion of the HFs management in the IPC team, however, this is lacking in most HFs in Nigeria. A positive relationship between the existence of organisational support and IPC has been identified in previous studies [24, 25]. This is because organisational involvement indicates the extent of value placed on staff care. This also indicates a higher likelihood for logistics and clinical supervision, as well as the development of training protocols adapted to the needs of the local HFs [26]. We also found that IPC focal persons (FPs) were lacking in each ward unit, as contrary to the standard practice. This explains a thorough lack of monitoring, assessment and feedback practice from HCWs in each unit of HFs. When IPC systems are strengthened, multifocal identification of staff needs to become possible, and then responsive actions can be taken to address such identified needs.

We also identified poor point of care risk assessment as a factor contributing to the spread of COVID-19 among HCWs. Ensuring triage, early recognition and source control isolating patients with suspected COVID-19 is the first IPC strategy to prevent or limit transmission in health care settings. Our findings however suggest that poor index of suspicion, failure to classify all patients as suspected cases and inadequate knowledge of patient triaging contribute to exposure of HCWs. Undisclosed history of exposure and delay in establishing a diagnosis further complicates HCWs exposure to COVID-19. A study in China established that HCWs who took training on standard precaution guidelines were more likely to always comply with IPC as compared to non-trained HCWs [13].

Findings from this study suggest that poor outbreak preparedness of the Nigerian government for the coronavirus contribute to the escalation in the number of infected HCWs. A likely explanation for this result is the lack of sufficient logistics put in place by the national government regarding the eventual importation of COVID-19. A survey conducted among HCWs during the EVD outbreak in Ghana showed ill preparedness of HFs to handle the cases [27]. In spite of this finding, the Nigerian Centre for Disease Control (NCDC) developed the Surveillance and Outbreak Response Management System (SORMAS) for case-based reporting early in the pandemic [28]. Similarly, the National Incident Coordination Centre (NICC) was established for gathering daily intelligence reports, and to ensure a well-coordinated outbreak response [29].

We also found that the deployment of non-critical care staff in the COVID-19 response at HFs could heighten the infection rates among HCWs. The reasons for this are as follows: Firstly, most of these HCWs provide regular health care services only. Secondly, the deployment of these HCWs directly into critical care for COVID-19 could increase their vulnerability to the infection. These factors were also stated in similar studies [4, 13]. Hence, improved outbreak preparedness for strengthening the national health system for events of future health emergencies was solicited among the respondents in this study.

Incentives and financial motivation serve to mitigate the risk with which HCWs are faced during the COVID-19 outbreak. We found in this study that a lack of incentives could increase the COVID-19 infection among HCWs. Unmotivated and tired HCWs are prone to make mistakes while discharging their duties. A study on the application of incentive schemes in health care acknowledges that financial incentives alone are not sufficient to retain and motivate staff, but also effectively improve care processes [13]. Thus, establishing a rewarding system for HCWs who consistently put their lives on the line should be advocated.

Poor political will and management support are factors that contribute to poor compliance to IPC in this study. A similar finding was corroborated in an Ethiopian study where management support in HFs positively impacted the compliance of HCWs with IPC [5]. This might be because political and HFs management authorities play key roles and are responsible for an improved access of necessary safety equipment to physicians, nurses and other cadres of HCWs. The active commitment of management authorities in HFs would also enhance investment in safe work environments for HCWs and patients. It is also obvious that, without the support of management teams in HFs, it would be difficult to renovate infrastructures which are suitable for infection control. Difficulties would also be experienced in the allocation of sufficient budget for IPC activities such as training of HCWs [4, 6]. Active political involvement would ensure the decentralisation of COVID-19 treatment centres, which would reduce both the workload of HCWs and the risk of contracting COVID-19 [5]. In addition, HFs management support could also help strengthen infection prevention activities by designing controlling mechanisms and taking corrective measures regarding non-compliant HCWs.

### Strengths and limitations

The findings in this study could have been limited by being carried out in only one state. The inclusion of only physicians and nurses could have concealed the experiences of other cadres of HCWs who were also frontline HCWs in the COVID-19 response. Despite these limitations, the in-depth interview method made use of in this study elicited robust information regarding the experiences of frontline HCWs in the COVID-19 response. This study also provides novel information on the experiences of frontline HCWs who are involved in the COVID-19 response in North-Central Nigeria. The qualitative research helped in the investigation of individual views and perceptions, and to identify strategies for the reduction of COVID-19 infection rates among HCWs. It further provides a solid background for developing concepts required for future quantitative research.

## Conclusion

This study reviewed the causes of COVID-19 infections among HCWs from the point of view of front-line HCWs in Kwara State Nigeria. The respondents were aware of their vulnerability to the COVID-19 infection, and the reasons attributed included poor knowledge of IPC measures for COVID-19, inadequate supply of PPE, poor political will and inadequate HFs management support. We recommend scale-up training on IPC measures particularly hand washing and use of PPE as well as the development of effective points of care risk assessment with high index of suspicion in HFs. In addition, improved political will and better involvement of HFs management teams in IPC systems is needed to reduce the risk for COVID-19 infection among HCWs.

## Data Availability

The findings in this study do not rely on any data or code.

## Supplementary material

For supplementary material accompanying this paper visit https://doi.org/10.1017/S0950268821000017. click here to view supplementary material

## References

[1] Ilesanmi OS and Afolabi AA (2020) Perception and practices during the COVID-19 pandemic in an urban community in Nigeria: a cross-sectional study. Peer Journal 8, e10038. 10.7717/peerj.10038 PMC7519720 33024646

[2] Chang D (2020) Protecting health-care workers from subclinical coronavirus infection. The Lancet Respiratory Medicine 8, e13. 32061333 10.1016/S2213-2600(20)30066-7 PMC7128440

[3] Zhong B-L (2020) Knowledge, attitudes, and practices towards COVID-19 among Chinese residents during the rapid rise period of the COVID-19 outbreak: a quick online cross-sectional survey. International Journal of Biological Sciences 16, 1745–1752. 32226294 10.7150/ijbs.45221 PMC7098034

[4] Chen Q (2020) Mental health care for medical staff in China during the COVID-19 outbreak. The Lancet Psychiatry 7, e15–e16. 32085839 10.1016/S2215-0366(20)30078-X PMC7129426

[5] Ilesanmi OS and Afolabi AA (2020) Time to move from vertical to horizontal approach in our COVID-19 response in Nigeria. SciMedicine 2, 28–29.

[6] Li L, Xv Q and Yang J (2020) COVID-19: the need for continuous medical education and training. The Lancet Respiratory Medicine 8, e23. doi: 10.1016/S2213-2600(20):30125-9. 32192586 PMC7104229

[7] Bazeyo W (2015) Ebola a reality of modern public health; need for surveillance, preparedness, and response training for HCWs and other multidisciplinary teams: a case for Uganda. Pan African Medical Journal 20, 404. 10.11604/pamj.2015.20.404.6159 PMC4524909 26301008

[8] Euro News. ‘At least 90,000 healthcare workers infected with COVID-19’, says nursing group. Available at https://www.euronews.com/2020/05/06/at-least-90-000-healthcare-workers-infected-by-covid-19-says-nursing-group (Accessed 3 July 2020).

[9] Ripples Nigeria. HCWs infected with COVID-19 in Nigeria now 812 —NCDC DG. Available at https://www.ripplesnigeria.com/health-workers-infected-with-covid-19-in-nigeria-now-812-ncdc-dg/ (Accessed 3 July 2020).

[10] Kim KH (2017) Middle East Respiratory syndrome coronavirus (MERS-CoV) outbreak in South Korea, 2015: epidemiology, characteristics and public health implications. Journal of Hospital Infection 95, 207–213. 10.1016/j.jhin.2016.10.008 PMC7114867 28153558

[11] Elkholy AA (2020) MERS-CoV infection among healthcare workers and risk factors for death: retrospective analysis of all laboratory-confirmed cases reported to WHO from 2012 to 2 June 2018. Journal of Infection and Public Health 13, 418–422. 31056437 10.1016/j.jiph.2019.04.011 PMC7102841

[12] Maunder R (2003) The immediate psychological and occupational impact of the 2003 SARS outbreak in a teaching hospital. Canadian Medical Association Journal 10, 1245–1251. PMC154178 12743065

[13] Liu Q (2020) The experiences of health-care providers during the COVID-19 crisis in China: a qualitative study. The Lancet Global Health 8, e790–e798. 32573443 10.1016/S2214-109X(20)30204-7 PMC7190296

[14] Ong M-S (2013) Communication interventions to improve adherence to infection control precautions: a randomized crossover trial. BMC Infectious Diseases 72, 1–9. 10.1186/1471-2334-13-72 PMC3599084 23388051

[15] Ong MS (2010) Coiera E: safety through redundancy: a case study of in-hospital patient transfers. Quality & Safety in Health Care 19, e32. 10.1136/qshc.2009.035972 20671076

[16] Colaizzi PF (1978) Psychological research as the phenomenologist views it In Valle RS and King M (eds), Existential-phenomenological Alternatives for Psychology. New York, NY: Oxford University Press, 48–71.

[17] Braun V and Clarke V (2006) Using thematic analysis in psychology. Qualitative Research in Psychology 3, 77–101.

[18] World Health Organization (2016) Personal protective equipment for use in a filovirus disease outbreak: Rapid advice guideline. Available at http://www.who.int/csr/resources/publications/ebola/personal-protective-equipment/en/ (Accessed 5 July 2020). 27929622

[19] Lee SM (2018) Psychological impact of the 2015 MERS outbreak on hospital workers and quarantined hemodialysis patients. Comprehensive Psychiatry 87, 123–127. 30343247 10.1016/j.comppsych.2018.10.003 PMC7094631

[20] Marjanovic Z, Greenglass ER and Coffey S (2007) The relevance of psychosocial variables and working conditions in predicting nurses’ coping strategies during the SARS crisis: an online questionnaire survey. International Journal of Nursing Studies 44, 991–998. 16618485 10.1016/j.ijnurstu.2006.02.012 PMC7094220

[21] World Health Organization (2011) Core Components for Infection Prevention and Control Programs: Assessment Tools for IPC Programs. Geneva, Switzerland: World Health Organization.

[22] Wang J, Zhou M and Liu F (2020) Reasons for healthcare workers becoming infected with novel coronavirus disease 2019 (COVID-19) in China. Journal of Hospital Infection 105(1), 100–101. doi: 10.1016/j.jhin.2020.03.002. PMC7134479 32147406

[23] Schwartz J, King C-C and Yen M-Y (2020) Protecting healthcare workers during the coronavirus disease 2019 (COVID-19) outbreak: lessons from Taiwan's severe acute respiratory syndrome response. Clinical Infectious Diseases 71(15), 858–860. doi: 10.1093/cid/ciaa255. 32166318 PMC7108122

[24] Ptaff KA (2014) Exploring new graduate nurse confidence in interprofessional collaboration: a mixed methods study. International Nursing Studies 51, 1141–1152. 10.1016/j.ijnurstu.2014.01.001 24486164

[25] Bookey-Bassett S (2017) Understanding inter-professional collaboration in the context of chronic disease management for older adults living in communities: a concept analysis. Journal of Advanced Nursing 73, 71–84. 27681818 10.1111/jan.13162

[26] Kebe NM (2020) Variables associated with interprofessional collaboration: a comparison between primary healthcare and specialized mental health teams. BMC Family Practice 21, 1–11. 31914942 10.1186/s12875-019-1076-7 PMC6950896

[27] Annan AA (2017) Health care workers indicate ill preparedness for ebola virus disease outbreak in Ashanti region of Ghana. BMC Public Health 546. doi: 10.1186/s12889-017-4474-6. PMC5461762 28587602

[28] Nigerian Centre for Disease Control (NCDC) (2019) 2018 NCDC Annual Report. [Online]. Available at https://ncdc.gov.ng/themes/common/files/annualreports/18803aba6209ada4ad84c8db76c22ea.pdf (Accessed 05 July 2020).

[29] Mustapha JO, Adedokun KA and Abdullahi IN (2020) Public health preparedness towards COVID-19 outbreak in Nigeria. Asian Pacific Journal of Tropical Medicine 13, 1–2.

