# Supplementary material for: Infection prevention and control during COVID-19 pandemic: realities from health care workers in a north central state in Nigeria
Source: Epidemiol Infect. 2021 Jan 7;149:e15. doi: 10.1017/S0950268821000017 (PMC7844182; doi:10.1017/S0950268821000017)
Supplement: Supplementary file 1 [file S0950268821000017sup001.docx]

**IPC: COVID-19 INFECTION AMONG HEALTH CARE WORKERS IN KWARA STATE**

**SECTION A: SOCIO DEMOGRAPHIC DATA**

1. Age: …………………………………
2. Sex: 1. Male 2. Female

3. Religion: 1. Christianity 3. Traditional

2. Islam 4. Others, please specify………………

4. Ethnicity: 1 Yoruba 3. Hausa

2. Ibo 4. Others, please specify………………….....

5. Highest level of education: ………………………………………………

6. Highest Medical qualification: …………………………………………….

7. Profession: 1. Doctor 2. Pharmacist 3. Nurse

4. Physiotherapist 5. Community Health Worker

6. Others, please specify…………………………..

8. Number of years in practice of profession: …………………………..

9. Cadre: ………………………………………………………………………

10. Grade level: ………………………………………………………………….

11. What type of health facility do you work?

1. Primary Health Care

2. Secondary Health Care/General Hospital

3. Tertiary Health Care/Teaching Hospital

4. COVID-19 Isolation Centre

5. Others (specify)……………………………………………………………….

ICE BREAKER

12. Are you aware of any health care worker infected with COVID-19 in Kwara State

1. Yes 2. No

13. If yes to question 12, how many staff do you think have been infected? _______

**SECTION B: INFECTION AMONG HEALTH CARE WORKERS**

14. What contributes to COVID-19 infection among health care workers in Kwara State?

15. Are there other factor that supports?

16. What have you tried to solve the problem of COVID-19 infection among health care workers in Kwara State?

17. What other solution can you suggest to solve the problem of COVID-19 infection among health care workers in Kwara State?

Thanks for your attention
